# The value of a prophage-borne defense system in phage–phage competition

**DOI:** 10.1371/journal.pcbi.1014739

**Published:** 2026-09-08

**Authors:** Yigal Meir, Ned S. Wingreen

**Affiliations:** 1 Department of Physics, Ben-Gurion University of the Negev, Beer Sheva, Israel; 2 Lewis-Sigler Institute for Integrative Genomics, Princeton University, Princeton, New Jersey, United States of America; 3 Department of Molecular Biology, Princeton University, Princeton, New Jersey, United States of America; BioInnovation Institute, DENMARK

## Abstract

Temperate phages that incorporate into their bacterial hosts’ genomes often encode defense systems that protect their hosts from superinfection by unrelated phages. Yet the evolutionary value of such defenses to the phage remains unclear. We present a minimal theoretical framework to quantify the selective advantage of a prophage-borne defense system in competition between temperate phages infecting the same bacterial host. The model reveals regimes in which a “defensive phage” can invade and persist despite growth costs, regimes of bistability, and others in which all phage types coexist due to a rock–paper–scissors-like dynamic between defensive, non-defensive, and defense-loss variants. Because defense systems can be non-transitive, true rock-paper-scissors relations can lead to persistent oscillations. These results identify simple conditions under which phage-encoded defense systems are evolutionarily stable, providing testable predictions for the prevalence and maintenance of these systems in natural microbial communities.

## Author summary

Bacteria are susceptible to their own viruses, called bacteriophages or simply phages. Temperate phages are able to integrate their DNA into bacterial genomes and persist as “prophages.” Surprisingly, many prophages encode defense systems that protect their bacterial host from infection by other phages. While such defenses are widespread in microbial genomes, it is not obvious why they benefit the prophage itself – especially because defense systems typically slow host growth or carry other costs. We developed a simple mathematical model to ask when a prophage-borne defense system is favored during competition between temperate phages infecting the same bacterial species. By focusing on long-term population dynamics, we identify conditions under which a “defensive” prophage can invade and persist, even if it reduces host growth by as much as 20%. If defense systems can be lost by mutation, a striking outcome emerges: defensive and non-defensive variants can stably coexist through a rock–paper–scissors-like interaction. Our results provide a quantitative framework for understanding why phage-encoded defense systems are so common in nature – they show that even costly defenses can be evolutionarily stable when phages frequently compete for the same hosts.

## Introduction

Temperate bacteriophages frequently encode defense systems that protect their bacterial hosts from infection by other phages, thereby indirectly promoting the persistence and transmission of the prophage itself [1–5]. These prophage-borne defenses span diverse molecular strategies and act at multiple stages of the infection cycle, from blocking adsorption or genome injection (classic superinfection exclusion) to targeting intracellular phage replication, transcription, or virion assembly [1,6]. Defenses may directly counter infection or, instead, activate abortive infection in which the prophage sacrifices the infected cell to prevent productive replication of the invader [3,5]. The range of such phage-borne defenses can be broad – systematic studies in multiple species showed that defenses can provide resistance to diverse incoming phages [1,2,5].

However, prophage-borne defenses are not free. Costs can arise from direct expression burdens, interference with core cellular processes, and tradeoffs associated with altered surface structures (e.g., transporters, pili, flagella, receptors) that reduce nutrient uptake or motility [1,7]. Some defense systems impose intrinsic risks of self-damage: restriction-modification systems can cause stochastic “autoimmunity” via self-restriction, triggering DNA damage responses and reducing fitness [8]. CRISPR-Cas immunity can likewise carry costs through maintenance and expression burdens and due to self-targeting spacers [9].

How large a cost can a defense system carry and still provide a net benefit to the prophage? Here, we present a simple theoretical framework that quantifies the selective value of a prophage-borne defense system in the specific context of competition between temperate phages infecting the same bacterial host. Bacteria–phage population dynamics have long been modeled using resource-explicit predator–prey or chemostat frameworks, including the classic work of Levin, Stewart, and Chao on lytic phages [10] and a later extension to study the advantages of lysogeny [11]. While works such as these have established a mathematical framework, a challenge in modeling competition among temperate phages is that spontaneous induction of prophages typically occurs at rates orders of magnitude slower than bacterial growth or phage-induced lysis. However, in environments where the timescale of phage adsorption is short compared to other relevant timescales such as bacterial division or lytic delay, it is mathematically consistent to assume newly released virions instantly find new hosts, obviating the need to track free phage populations. By assuming this separation of timescales, we are able to efficiently model interactions among lysogens over many rounds of induction. This simplified approach is particularly appropriate for modeling the evolutionary stability of phage-borne defense systems, as it focuses on the long-term dynamics that emerge from the trade-offs between prophage-borne protection and its costs. Our model reveals parameter regimes under which defense systems are predicted to be stably maintained, thereby offering predictions for the prevalence of these systems in natural bacterial populations.

## Results

### Carrying a defense-system has competitive value to a prophage

**Fig 1:**
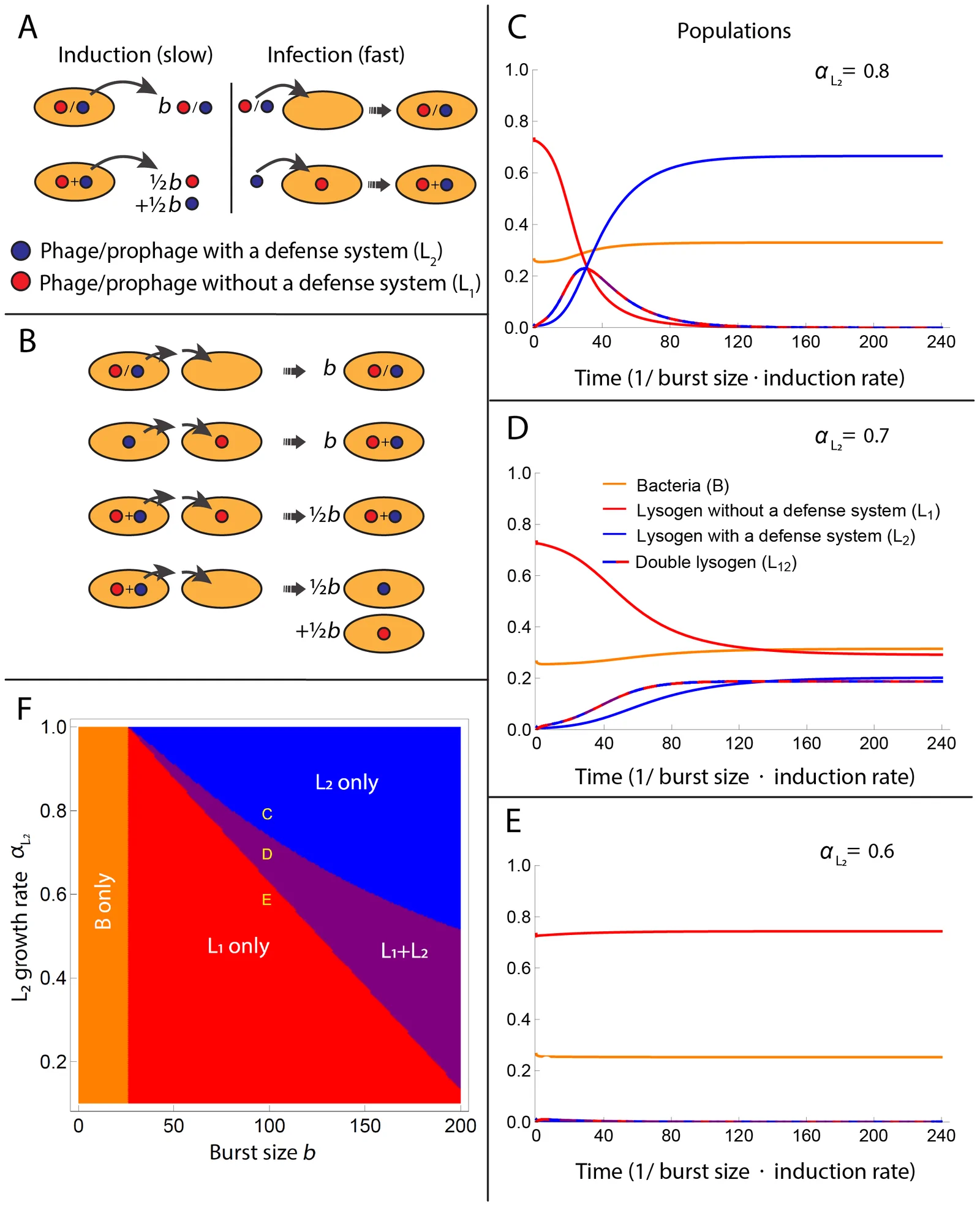
A phage carrying a costly anti-phage defense system can successfully compete against other phage. (A,B) Schematics of model elements: (A) Each lysogen ($L_{1}$, $L_{2}$, and the double lysogen $L_{12}$) induces at a constant, low rate *k* (left), with the $L_{12}$ lysogen releasing an equal number of $L_{1}$-type and $L_{2}$-type phage virions. The resulting burst of *b* released virions rapidly attacks all types bacteria in proportion to their population sizes, but only some attacks lead to successful infections: (1) any invasion of susceptible bacteria (top right), (2) invasion of the defenseless lysogen $L_{1}$ by an $L_{2}$-type virion, resulting in a double lysogen $L_{12}$ (bottom right). (B) The two processes in (*A*) are combined into a single process (double arrows). The first line, for example, describes $L_{1}$/ $L_{2}$ lysogens inducing and immediately infecting susceptible bacteria, resulting in *b* copies of the same lysogen. (C-E) Time courses of invasion. A steady-state system of susceptible bacteria (B) and a lysogen ($L_{1}$) is invaded by a lysogen ($L_{2}$) with an anti-phage defense system; different outcomes are possible depending on the growth rate of $L_{2}$: (C) $L_{2}$  drives $L_{1}$  extinct for $\alpha_{L_{2}}=0.8$, (*D*) coexistence of $L_{1}$  and $L_{2}$  for $\alpha_{L_{2}}=0.7$, (E) $L_{2}$  dies out for $\alpha_{L_{2}}=0.6$. (F) Phase diagram of outcomes with respect to growth rate of $L_{2}$  and burst size; letters C, D, E indicate corresponding $\alpha_{L_{2}}$ and *b* values for those panels. Parameters for plots: growth rates $\alpha_{B}=\alpha_{L_{1}}=1$ and $\alpha_{L_{12}}=\alpha_{L_{2}}$, bacteria source *S*=0.001, burst size *b* = 100, dilution rate δ=0.005, and induction rate $k=4\times 10^{-5}$.

To quantify the value of a phage-borne defense system in phage-phage competition, we consider a competition between two temperate phages that predate on the same bacterial host, with the main processes shown schematically in Fig 1A and 1B. One of the phages carries a defense system that makes its lysogens immune to infection by the other phage. (Phages of each type are assumed to be immune to superinfection by the same type.) To focus on the role of the defense system, we make several simplifying assumptions: (1) We consider a chemostat-like setting with a steady supply rate of susceptible bacteria, and a constant dilution (loss) rate of all constituents. (2) We do not track free phage particles, instead assuming that these rapidly find bacterial targets. To our knowledge, this useful approximation for modeling lysogen population dynamics is new to the field. (3) We do not explicitly simulate nutrients, but rather assume a fixed overall carrying capacity for bacteria. With these assumptions, the non-dimensionalized dynamical equations for the system are (the full set of dimensional equations is detailed in Materials and Methods, subsection M1):


$$\begin{array}{c} \begin{array}{c} \frac{dB}{dt}=S+B(1-P_{tot})-\delta B-bk(L_{1}+L_{2}+L_{12})B/P_{tot}, \end{array} \end{array}$$ (1)

$$\begin{array}{c} \begin{array}{cc} \frac{dL_{1}}{dt} & =L_{1}(1-P_{tot})-\delta L_{1}+bk(L_{1}+\frac{1}{2}L_{12})B/P_{tot}\\ & \;-bk(L_{2}+\frac{1}{2}L_{12})L_{1}/P_{tot}-kL_{1}, \end{array} \end{array}$$ (2)

$$\begin{array}{c} \begin{array}{c} \frac{dL_{2}}{dt}=\alpha_{L_{2}}L_{2}(1-P_{tot})-\delta L_{2}+bk(L_{2}+\frac{1}{2}L_{12})B/P_{tot}-kL_{2}, \end{array} \end{array}$$ (3)

$$\begin{array}{c} \begin{array}{c} \frac{dL_{12}}{dt}=\alpha_{L_{2}}L_{12}(1-P_{tot})-\delta L_{12}+bk(L_{2}+\frac{1}{2}L_{12})L_{1}/P_{tot}-kL_{12}, \end{array} \end{array}$$ (4)

with $P_{tot}=B+L_{1}+L_{2}+L_{12}$, where the populations of the various strains are *B* for susceptible bacteria, *L*_1_, for the lysogen without a defense system, *L*_2_, for the lysogen with a defense system, and *L*_12_, for the double lysogen. The first term on the right-hand side of Eq. 1 describes the influx of susceptible bacteria at a constant source rate *S*. The second term in (1), along with the first terms in (2–4), describes the replicative growth of the susceptible bacteria and the different lysogens up to a carrying capacity, defined as unity (which sets the scale for the populations). Here we assume that the growth rate of $L_{1}$ is the same as that of the susceptible bacteria [1], while only $L_{2}$ and $L_{12}$ have reduced growth rates $\alpha_{L_{2}}$ due to the cost of the defense system (we also tested an intermediate lower growth rate for the defenseless lysogen $L_{1}$, which did not lead to any qualitative changes in the results). The next terms describe decreases in the populations due to dilution and death at a rate δ. The last terms in Eqs. (2)–(4) describe induction at a rate *k*. The 3rd terms in (2) and (3) describe the increase in the lysogen population due to induction, with burst size *b*, immediately followed by infection of susceptible bacteria, while the 4th term in (1) describes the corresponding decrease in the susceptible bacteria population. Lastly, the 4th term in (2) and the 3rd term in (4) describe the conversion of $L_{1}$ to $L_{12}$ due to induction plus infection by either $L_{2}$ or $L_{12}$. The inverse process is not allowed due to the defense system of $L_{2}$. Later, we also consider an alternative case in which the defense system is abortive, namely $L_{2}$ dies when invaded by $L_{1}$. For simplicity, we assume, here and in the following that when a double lysogen induces equal numbers of the two prophage particles are produced.

Equations 1–4 lead to distinct outcomes depending on the cost of the defense system. In Fig 1C – 1E, we show timecourses for invasions by a small population of $L_{2}$  into steady-state systems of B and $L_{1}$. For the smallest cost of the defense system (highest $\alpha_{L_{2}}$), the invader drives $L_{1}$  to extinction; for intermediate cost, all strains coexist; and for the largest cost (lowest $\alpha_{L_{2}}$) the invasion fails. These results are summarized in the phase diagram in Fig 1F. (In the orange region, burst sizes are too small for lysogens to survive – the steady supply of susceptible bacteria *B* exhausts the system’s carrying capacity).

### Prophage-borne abortive infection leads to bistability

**Fig 2:**
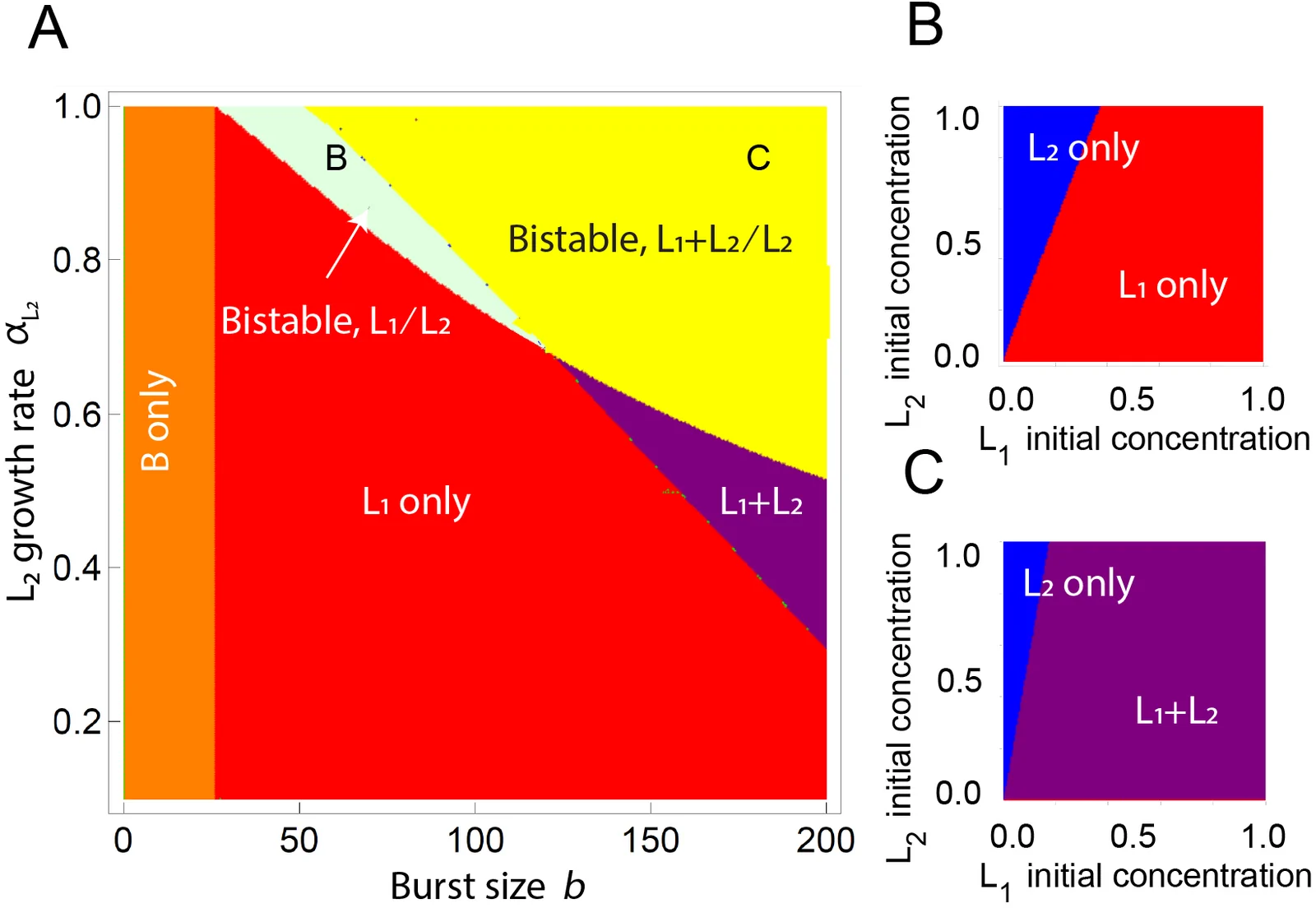
An abortive infection defense system is less valuable to a phage, and leads to population-level bistability. $L_{2}$ contains an abortive infection defense system, so that $L_{2}$ dies without a burst when infected by $L_{1}$ virions. (A) Phase diagram of outcomes with respect to growth rate of $L_{2}$  and burst size. Compared to Fig 1F, the region of $L_{2}$ dominance is decreased, and regions of bistability appear. Letters B, C, indicate the values $\alpha_{L_{2}}=0.95$ and *b* = 60,180 used for those panels, respectively. (B,C) Dependence of bistable outcomes on initial populations of $L_{1}$ and $L_{2}$ for white region (B) or purple region (C). Parameters for plots: growth rates $\alpha_{B}=\alpha_{L_{1}}=1$ and $\alpha_{L_{12}}=\alpha_{L_{2}}$, initial bacteria population *B*(*t* = 0) = 0.3, bacteria source *S* = 0.001, dilution rate δ=0.005, and induction rate $k=4\times 10^{-5}$.

Abortive infection is a qualitatively distinct alternative to the type of defense considered above. In contrast to defenses that prevent adsorption, genome entry, or intracellular replication while preserving the infected lysogen, abortive infection systems protect the surrounding clonal population by causing infected cells to die or arrest growth before the invading phage can complete its lytic cycle. Such systems are widespread and modern mechanistic studies have shown that toxin–antitoxin and related systems can function as abortive infection modules [12]. Importantly for the present study, abortive infection can also be prophage-borne. For example, the prophage-encoded BstA family mediates abortive infection against competing phages while cognate self-immunity protects the prophage that encodes the system [3]. To capture this biologically distinct mode of defense, we modified the model so that infection of an $L_{2}$ lysogen by virions released from $L_{1}$ does not generate a double lysogen $L_{12}$. Instead, $L_{2}$ cells infected by $L_{1}$-type virions are removed from the population, representing abortive death of the infected lysogen together with failure of the invading phage to establish a productive infection. We assume that $L_{12}$ is immune to $L_{1}$ virions via $L_{1}$’s superinfection exclusion, and thus the abortive infection system is not activated. (See Materials and Methods, section M2 for the corresponding modified equation for $L_{2}$.) As shown in Fig 2A, the death of $L_{2}$ lysogens upon infection by $L_{1}$ virions increases the region of $L_{1}$ dominance. More surprisingly, abortive infection also leads to regions of bistability where the final outcome depends on the initial ratio of $L_{1}$ to $L_{2}$, a prediction that could be tested experimentally. Fig 2B and 2C show the borderline ratio between the lysogens, which is independent of the initial bacteria population.

To gain insight into the regimes of bistability which occur when the growth rate of $L_{2}$ is high, it is helpful to consider the dynamics of the difference between $L_{1}$ and $L_{2}$ populations in the limit where $\alpha_{L_{2}}=1$. In this limit, subtracting the modified equation for *dL*_2_/*dt* (see Materials and Methods, section M2) from Eq. 2 for *dL*_1_/*dt* yields


$$\begin{array}{c} \frac{d(L_{1}-L_{2})}{dt}=[(1-P_{tot})-\delta +bkB/P_{tot}-bk\frac{1}{2}L_{12})/P_{tot}-k](L_{1}-L_{2}). \end{array}$$ (5)

The term that multiplies $\left(L_{1}-L_{2}\right)$ is typically positive, which means that when *L*_1_ = *L*_2_, the dynamics of $L_{1}-L_{2}$ is unstable, i.e., small positive values become more positive, while small negative values become more negative. Such divergent behavior is characteristic of bistability. One point to note is that in this limit, Eq. 2 for *L*_1_ and the modified Eq. 3 for *L*_2_ are actually perfectly symmetric – so why is there an asymmetry in the bistable solutions at large burst size, with either only $L_{2}$ surviving or coexistence of $L_{1}$ and $L_{2}$? To understand this asymmetry, consider the dynamics of *L*_12_ around the two steady states where only $L_{1}$ or only $L_{2}$ exist: in either case invasion by a small amount of the competing lysogen leads to creation of $L_{12}$ lysogens via the process of $L_{2}$ lysogens inducing and converting $L_{1}$ lysogens to $L_{12}$. For the $L_{2}$-only state, the further induction of these $L_{12}$ lysogens has little effect because their $L_{2}$-type virions can only convert the small population of $L_{1}$ lysogens. By contrast, for the $L_{1}$-only fixed point, the $L_{2}$-type virions from the induced $L_{12}$ lysogens target the initially large population of $L_{1}$ lysogens, leading to an exponential increase of the $L_{12}$ population. This is the key to the asymmetry in outcomes whereby the $L_{2}$-only state is stable against invasion, while the $L_{1}$-only state is unstable against invasion.

### Consequences of mutational loss of a prophage-borne defense system

**Fig 3:**
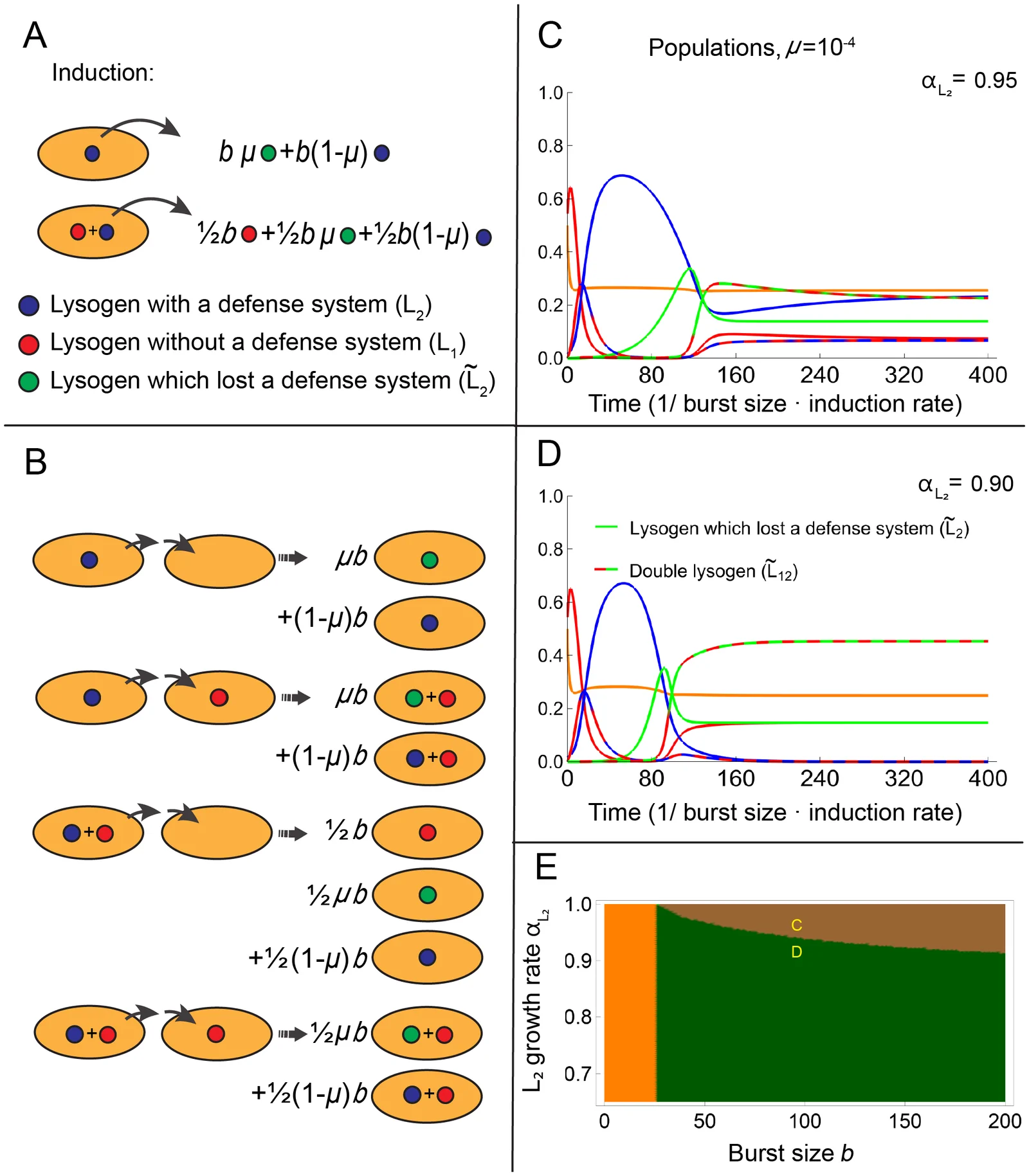
A finite loss rate of a phage-borne anti-phage defense system can lead to multi-strain coexistence. (A,B) Schematics of additional model elements: (A) Upon infection, the phage that creates lysogen $L_{2}$  has a small mutation probability μ to lose its defense system (defenseless version denoted by a tilde). (B) Possible infection processes that result in defense system loss. While the phage that creates lysogen $L_{1}$  can invade lysogen ${\tilde{L}}_{2}$  and vice versa, the phages that create $L_{2}$  and ${\tilde{L}}_{2}$  are of the same type and so cannot invade each other. (C,D) Time courses of invasion (line colors not appearing in the legend are the same as in Fig 1). A steady-state system of susceptible bacteria (B) and a lysogen ($L_{1}$) is invaded by a lysogen ($L_{2}$) with an anti-phage defense system that can be lost; different outcomes are possible depending on the growth rate of $L_{2}$: (C) coexistence of all strains for $\alpha_{L_{2}}=0.95$, (D) loss of $L_{2}$  but coexistence of all defenseless strains for $\alpha_{L_{2}}=0.9$. (E) Phase diagram of outcomes with respect to growth rate of $L_{2}$  and burst size; letters C, D indicate corresponding $\alpha_{L_{2}}$ and *b* values for those panels. Parameters for plots as in Fig 1, with $\mu =10^{-4}$; defenseless strains all have growth rate 1.

Fig 1F suggests that carrying a defense system can be quite advantageous for a phage. However, it is worth noting that if a phage-borne defense system has a small enough cost to drive competing phages to extinction, then the defense system might make itself unnecessary. Does this mean that phages should eventually lose their defense systems? To address this question, we allow for the defense system to be lost at a mutation rate μ during a new infection, as shown in Fig 3A and 3B. This adds two new types of lysogen ${\tilde{L}}_{2}$  and ${\tilde{L}}_{12}$  which carry versions of the temperate phage that have lost the defense system, and so do not pay a growth cost. For example, the population of ${\tilde{L}}_{2}$  obeys


$$\begin{array}{c} \begin{array}{cc} \frac{d{\tilde{L}}_{2}}{dt} & ={\tilde{L}}_{2}(1-P_{tot})-\delta {\tilde{L}}_{2}-bk(L_{1}+\frac{1}{2}L_{12}){\tilde{L}}_{2}/P_{tot}\\ & \;+bk[({\tilde{L}}_{2}+\frac{1}{2}{\tilde{L}}_{12})+\mu (L_{2}+\frac{1}{2}L_{12})]B/P_{tot}. \end{array} \end{array}$$ (6)

In short, we now allow mutations from an $L_{2}$ or $L_{12}$ lysogen into a phage without a defense system at a rate μ, and infection of ${\tilde{L}}_{2}$ lysogens by $L_{1}$ to make a double lysogen ${\tilde{L}}_{12}$ (see Material and Methods, subsection M3 for the full set of equations). With these additions, we repeated the invasion simulations and found a striking outcome: for a small enough cost of the defense system, all strains now coexist (Fig 3C). For a higher cost, the two lysogens without defense systems, $L_{1}$  and ${\tilde{L}}_{2}$, coexist symmetrically (Fig 3D), as they have identical characteristics. These results are summarized in the phase diagram in Fig 3E.

What is the origin of the state of complete coexistence? We attribute this state to a rock-paper-scissors-like relation among the three phages: As shown in Fig 3, $L_{2}$  outcompetes $L_{1}$. But now ${\tilde{L}}_{2}$  outcompetes $L_{2}$, since the former pays no cost for defense, and by assumption these two phages of the same basic type cannot invade each other. Finally, $L_{1}$  and ${\tilde{L}}_{2}$  coexist since they are effectively identical competitors.

### Rock-paper-scissors dynamics among competing lysogens

**Fig 4:**
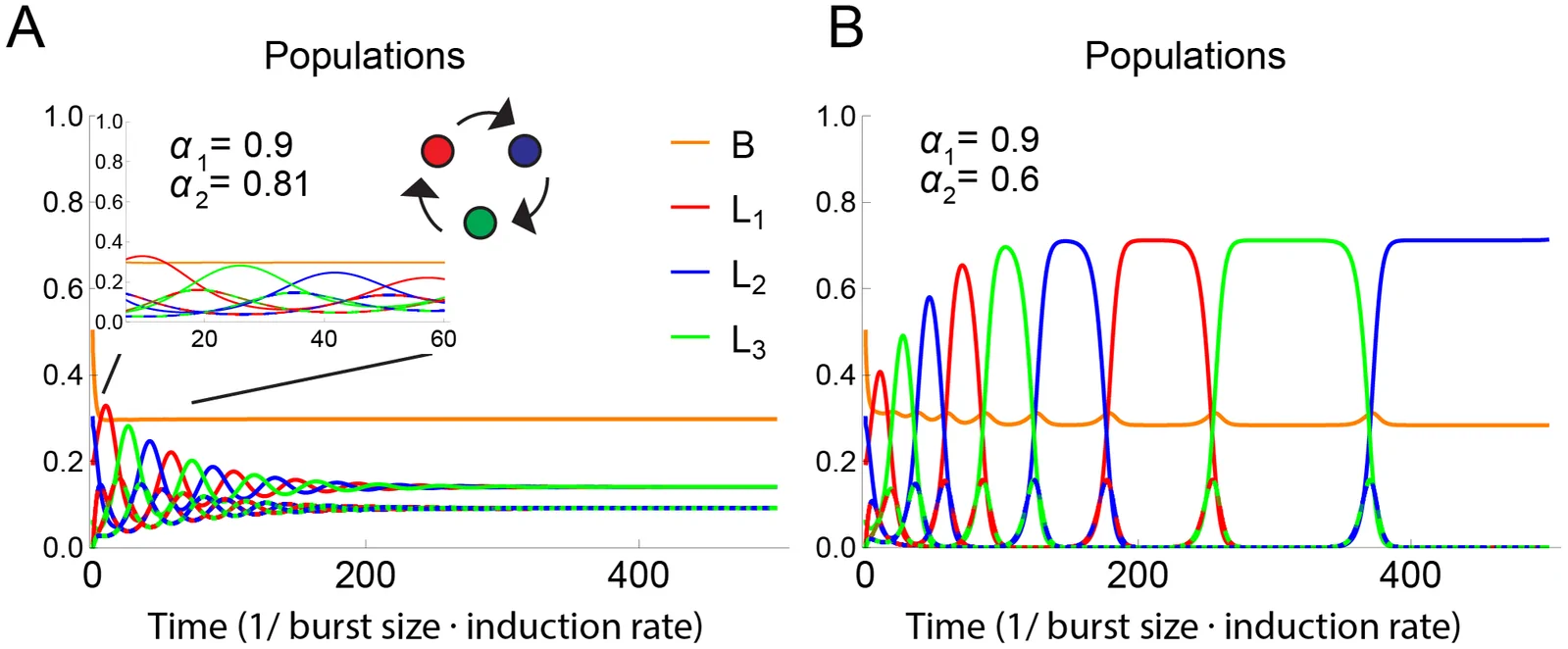
Phage-borne defense systems can lead to a rock-paper-scissors dynamic among lysogens. (A,B) Time courses of a system of three temperate phages carrying non-transitive defense systems: (*Inset*) $L_{1}$ is immune to $L_{2}$, $L_{2}$ is immune to $L_{3}$, and $L_{3}$ is immune to $L_{1}$. All double lysogens are present at low populations, but the triple lysogen is not possible. (A) Growth rates of single lysogens $\alpha_{1}=0.9$ and double lysogens $\alpha_{2}=0.81$ lead to slowing decaying oscillations to a coexistence fixed point. (B) Growth rates of single lysogens $\alpha_{1}=0.9$ and double lysogens $\alpha_{2}=0.6$ lead to persistent rock-paper-scissors oscillations with an increasing period. Other parameters for plots as in Fig 1.

Can phage-borne defense systems lead to true rock-paper-scissors dynamics? Defense systems can be non-transitive, so we simulated a system of three temperate phages with cyclic immunity, but otherwise identical parameters (see Materials and Methods, subsection M4 for the full set of equations). As shown in Fig 4, two distinct outcomes occur depending on the growth rates of the single and double lysogens (triple lysogens can’t occur): As shown in Fig 4A, if the double lysogen growth rate is high enough, the system shows damped rock-paper-scissors oscillations, but eventually relaxes to a coexisting fixed point. However, as seen in Fig 4B for a lower growth rate of the double lysogens, the oscillations persist, becoming longer in period but remaining large in amplitude.

## Discussion and conclusion

In summary, we have shown that it can be highly beneficial for a temperate phage to carry a defense system against competing phage. In a simple model of two competing phages, a defensive phage with a ~20% lower growth rate can still drive a competitor to extinction (for typical burst sizes of ~100 virions). However, in nature, the advantage of carrying a defense system will depend both on the frequency with which multiple types of phages compete for the same bacterial host, and the effective range of the defense system. Since in principle fitness advantages as small as $1/N_{e}$ can fix in bacterial populations, where effective population sizes $N_{e}$ range from 10^6^ to 10^8^ or more [13], even occasional competition between phages could still favor phage carrying their own defense systems. This is consistent with the observation of many prophage-borne defenses in bacterial genomes [1,2,5].

An interesting question is where do bacteria get their phage defense systems from? Many of these systems reside in “defense islands”, which contain multiple defenses [14,15], and the contents of these islands are highly variable between closely related strains [16]. However, defenses are also observed scattered throughout bacterial genomes [17,18]. Moreover, similar systems are found among distantly related bacteria, implying that the defenses move among species via horizontal gene transfer. Our analysis suggests that temperate phage may be a significant contributor to this movement of defenses.

Some natural extensions of our model would include competition between defensive temperate phages and lytic phages or phages carrying counter-defense systems. We have also assumed that the defense system is absolute, whereas defenses may only be partial or may be regulated, e.g., by quorum sensing [19,20]. In natural settings, the competition among lysogens may also be spatially structured (our lysogen-only representation may still be useful for spatiotemporal modeling under the assumption that free virions quickly find targets). These scenarios and settings will quantitatively change competitive outcomes, but are not expected to change the overall advantage of phage-borne defenses. Indeed, a recent theoretical study of competition between superinfection-exclusion-positive or -negative lytic phages came to a similar conclusion regarding the substantial value of such a phage-borne defense system [21].

From an experimental perspective, our model of serial dilutions with addition of new susceptible bacteria is readily realizable. The main limitation is that we have not included adaptive mutations of either host or phage, which would eventually occur. Careful choices of bacteria, phage, and nutrients could minimize the development of bacterial resistance, e.g., by including key nutrients that require transport via phage receptors. We hope our work will inspire further consideration of the ecology and evolution of phage-borne defense systems.

## Materials and methods

### M1 Dimensionful equations

The dimensionful version of Eqs. (1)–(4) are


$$\begin{array}{cc} \frac{dB^{*}}{dt^{*}} & =S^{*}+\alpha_{B}^{*}B^{*}(1-P_{tot}^{*}/\kappa )-\delta^{*}B^{*}-bk^{*}(L_{1}^{*}+L_{2}^{*}+L_{12}^{*})B^{*}/P_{tot}^{*},\\ \frac{dL_{1}^{*}}{dt^{*}} & =\alpha_{L_{1}}^{*}L_{1}^{*}(1-P_{tot}^{*}/\kappa )-\delta^{*}L_{1}^{*}+bk^{*}(L_{1}^{*}+\frac{1}{2}L_{12}^{*})B^{*}/P_{tot}^{*}-bk^{*}(L_{2}^{*}+\frac{1}{2}L_{12}^{*})L_{1}^{*}/P_{tot}^{*}-k^{*}L_{1}^{*},\\ \frac{dL_{2}^{*}}{dt^{*}} & =\alpha_{L_{2}}^{*}L_{2}^{*}(1-P_{tot}^{*}/\kappa )-\delta^{*}L_{2}^{*}+bk^{*}(L_{2}^{*}+\frac{1}{2}L_{12}^{*})B^{*}/P_{tot}^{*}-k^{*}L_{2}^{*},\\ \frac{dL_{12}^{*}}{dt^{*}} & =\alpha_{L_{12}}^{*}L_{12}^{*}(1-P_{tot}^{*}/\kappa )-\delta^{*}L_{12}^{*}+bk^{*}(L_{2}^{*}+\frac{1}{2}L_{12}^{*})L_{1}^{*}/P_{tot}^{*}-k^{*}L_{12}^{*}, \end{array}$$

with $P_{tot}^{*}=B^{*}+L_{1}^{*}+L_{2}^{*}+L_{12}^{*}$, and all starred parameters are dimensionful. In this work, we chose $\alpha_{L_{1}}^{*}=\alpha_{B}^{*}$ and $\alpha_{L_{12}}^{*}=\alpha_{L_{2}}^{*}$. Scaling all concentrations by the carrying capacity κ, and defining $t\equiv \alpha_{B}^{*}t^{*},S\equiv S^{*}/(\kappa \alpha_{B}^{*}),\delta \equiv \delta^{*}/\alpha_{B}^{*},k\equiv k^{*}/\alpha_{B}^{*}$ and $\alpha_{L_{2}}=\alpha_{L_{12}}\equiv \alpha_{L_{2}}^{*}/\alpha_{B}^{*}$, we arrive at the dimensionless equations quoted in the text.

### M2 Equations for the abortive infection defense system

For the abortive infection defense system, $L_{2}$ is lost upon being invaded by $L_{1}$, leading to an additional loss term to Eq. 3:


$$\begin{array}{c} \frac{dL_{2}}{dt}=\alpha_{L_{2}}L_{2}(1-P_{tot})-\delta L_{2}+bk(L_{2}+\frac{1}{2}L_{12})B/P_{tot}-bk(L_{1}+\frac{1}{2}L_{12})L_{2}/P_{tot}-kL_{2}. \end{array}$$

Eqs. (1), (2), and (4) are unchanged.

### M3 Equations allowing for mutational loss of defense system

The full set of dimensionless equations, which allows for mutations that lead to a loss of a defense system, is


$$\begin{array}{c} \begin{array}{cc} \frac{dB}{dt} & =S+B\;\left(1-\tilde{P}\right)-\delta B-\;bk\;\frac{(L_{1}+L_{2}+{\tilde{L}}_{2}+L_{12}+{\tilde{L}}_{12})B}{\tilde{P}},\\ \frac{dL_{1}}{dt} & =L_{1}\;\left(1-\tilde{P}\right)-\delta L_{1}+bk\;\frac{(L_{1}+L_{12}/2+{\tilde{L}}_{12}/2)B}{\tilde{P}}-bk\;\frac{L_{1}(L_{2}+{\tilde{L}}_{2}+L_{12}/2+{\tilde{L}}_{12}/2)}{\tilde{P}}-kL_{1},\\ \frac{dL_{2}}{dt} & =\alpha_{L_{2}}L_{2}\;\left(1-\tilde{P}\right)-\delta L_{2}+bk\;\frac{(1-\mu )(L_{2}+L_{12}/2)B}{\tilde{P}}-kL_{2},\\ \frac{d{\tilde{L}}_{2}}{dt} & ={\tilde{L}}_{2}\;\left(1-\tilde{P}\right)-\delta {\tilde{L}}_{2}+bk\;\frac{(\mu L_{2}+{\tilde{L}}_{2}+\mu L_{12}/2+{\tilde{L}}_{12}/2)B}{\tilde{P}}-bk\;\frac{{\tilde{L}}_{2}(L_{1}+L_{12}/2+{\tilde{L}}_{12}/2)}{\tilde{P}}-k{\tilde{L}}_{2},\\ \frac{dL_{12}}{dt} & =\alpha_{L_{2}}L_{12}\;\left(1-\tilde{P}\right)-\delta L_{12}+bk\;\frac{(1-\mu )(L_{2}+L_{12}/2)L_{1}}{\tilde{P}}-kL_{12},\\ \frac{d{\tilde{L}}_{12}}{dt} & ={\tilde{L}}_{12}\;\left(1-\tilde{P}\right)-\delta {\tilde{L}}_{12}+bk\;\frac{(L_{1}+L_{12}/2+{\tilde{L}}_{12}/2){\tilde{L}}_{2}}{\tilde{P}}+bk\;\frac{(\mu L_{2}+{\tilde{L}}_{2}+\mu L_{12}/2+{\tilde{L}}_{12}/2)L_{1}}{\tilde{P}}-k{\tilde{L}}_{12}, \end{array} \end{array}$$ (7)

with $\tilde{P}=B+L_{1}+{\tilde{L}}_{2}+L_{2}+L_{12}+{\tilde{L}}_{12}.$

### M4 Equations for the rock-paper-scissors model

The full set of the 3-phage rock-paper-scissors system that led to Fig 4 is given by


$$\begin{array}{c} \begin{array}{c} \dot{B}=S+B(1-P_{tot})-\delta B-bk\;\frac{(P_{tot}-B)B}{P_{tot}}, \end{array} \end{array}$$ (8)

$$\begin{array}{c} \begin{array}{c} {\dot{L}}_{1}=\alpha_{L1}L_{1}(1-P_{tot})-\delta L_{1}+bk\;\frac{\left(L_{1}+\frac{1}{2}L_{12}+\frac{1}{2}L_{13}\right)B}{P_{tot}}-bk\;\frac{L_{1}\left(L_{3}+\frac{1}{2}L_{13}+\frac{1}{2}L_{23}\right)}{P_{tot}}-kL_{1}, \end{array} \end{array}$$ (9)

$$\begin{array}{c} \begin{array}{c} {\dot{L}}_{12}=\alpha_{L12}L_{12}(1-P_{tot})-\delta L_{12}+bk\;\frac{\left(L_{1}+\frac{1}{2}L_{12}+\frac{1}{2}L_{13}\right)L_{2}}{P_{tot}}-kL_{12}, \end{array} \end{array}$$ (10)

with the equations for $L_{2},L_{3},L_{13}$ and *L*_23_ obtained by cycling the indices 1→2→3→1. Here $P_{tot}=B+L_{1}+L_{2}+L_{3}+L_{12}+L_{13}+L_{23}$. To generate Fig 4 we used *b* = 100 and $\alpha_{L1}=\alpha_{L2}=\alpha_{L3}=\alpha_{1}$, and $\alpha_{L12}=\alpha_{L23}=\alpha_{L23}=\alpha_{2}$, where the values of $\alpha_{1}$ and $\alpha_{2}$ are quoted in the caption. All other parameters are the same as in Fig 3.

All sets of equations were simulated using Mathematica, which was also used to generate the plots.
