# Supplementary material for: The value of a prophage-borne defense system in phage–phage competition
Source: PLoS Comput Biol. 2026 Sep 8;22(9):e1014739. doi: 10.1371/journal.pcbi.1014739 (PMC13577574; doi:10.1371/journal.pcbi.1014739)
Supplement: Supplementary file 1 [file pcbi.1014739.s001.pdf]

REVIEW for submission PCOMPBIOL-D-26-00504 to PLOS Computational Biology

"The Value of a Prophage-Borne Defense System in Phage–Phage Competition"

by Yigal Meir and Ned S. Wingreen

The authors present a theoretical framework to study competition between temperate bacteriophages that infect the same bacterial host, with a particular focus on the evolutionary role of prophage-borne defense systems. The manuscript combines biological motivation with a relatively simple dynamical model formulated as a system of ordinary differential equations describing the interactions between susceptible bacteria and a few combinations of lysogens.

Personally, I appreciate short and concise articles, and this manuscript largely falls into that category, which made it relatively straightforward to read and review. At the same time, the presentation might benefit from a slightly more detailed discussion of the dynamical role (biological interpretation) of the individual terms in the ODE system. E.g., it would be very helpful in understanding the structure of the model if the authors were to explain the process transitions using one of the classical methods, e.g., compartmental transitions. I feel that mass action kinetics are also applicable here, as the transitions in Fig. 1B can be interpreted as chemical-like reactions.

I should also note that my own background is primarily on the mathematical side, and I am not a specialist in the biological aspects of bacteriophage systems. Nevertheless, I made an effort to follow the biological motivation and the modeling assumptions throughout the manuscript.

While going through the equations carefully, I noticed a few potential typos in Eq. (6). In Section 1, I outline this point in more detail.

In addition, I did not identify previously published work by the authors that would substantially overlap with the present manuscript.

Minor comments and suggestions

Although I did not systematically check the manuscript from a spelling or copy-editing perspective, I have a few minor remarks. Authors may decide, at their best assessment, whether or not to accept these recommendations.

- Fig 1B. It would be convenient to emphasize that the transition in Fig 1B encode the six possible pairs of the transition from Fig 1A (an induction + an infection).
- Fig 1F. is a very fine grained vector graphics, and it requires a long time to load. Consider whether it is worth generating a high-quality rasterized image instead.
- It was an even longer time before I realized that "SI Appendix" refers to "Supporting Information." I though I missed to download an additional supplementary document. I advise a more straightforward reference to this section in the main text.
- The lines of Eq. (6) are too long. As far as I know, the following LaTeX environment can expand the equations along the whole width of the page:

```
\begin{adjustwidth}{-2.25in}{0in}column.
    \begin{equation} ....
    \end{equation}
\end{adjustwidth}
```

1 Equations allowing for mutations and loss of defense systems

For clarity, I restate the equation in the form that seems to follow naturally from the processes described in the model, and I provide a schematic illustrations in Figure 1 that explain how the relevant terms arise. My intention

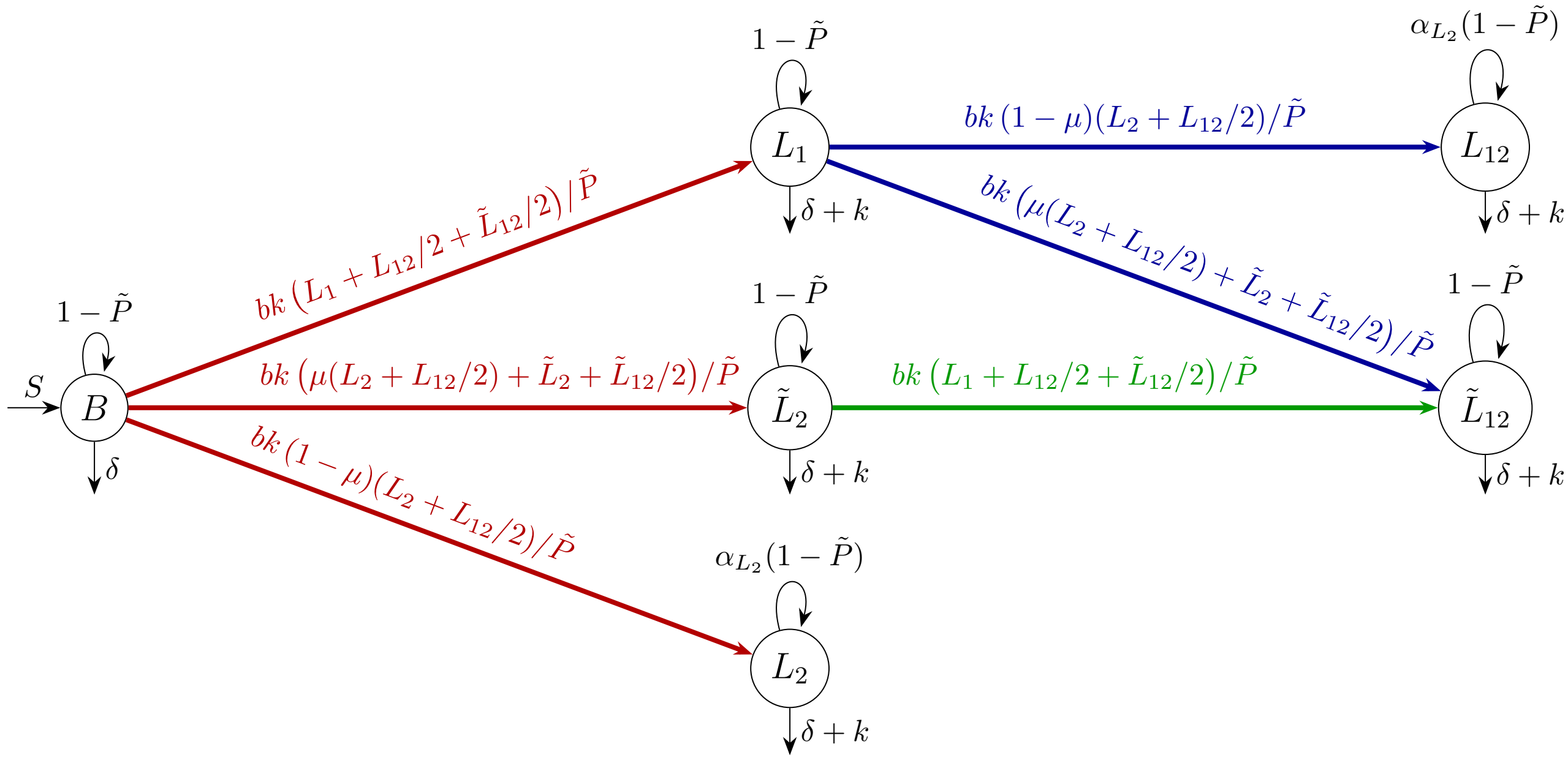

Figure 1: Compartmental transitions of the phage-phage competition. The infection mechanisms can be written as compartmental transitions, which preserves the overall population.

here is not to challenge the overall conclusions of the manuscript, but simply to clarify a point that may help make the mathematical formulation more transparent.

$$\dot{B} = S + B(1-\tilde{P}) - \delta B - \boxed{bk\left(L_1 + L_2 + \tilde{L}_2 + L_{12} + \tilde{L}_{12}\right)\frac{B}{\tilde{P}}}, \tag{1a}$$

$$\dot{L}_1 = L_1(1-\tilde{P}) - \delta L_1 + \boxed{bk\left(L_1 + L_{12}/2 + \tilde{L}_{12}/2\right)\frac{B}{\tilde{P}}} - \boxed{bk\left(L_2 + \tilde{L}_2 + L_{12}/2 + \tilde{L}_{12}/2\right)\frac{L_1}{\tilde{P}}} - kL_1, \tag{1b}$$

$$\dot{L}_2 = \alpha_{L_2} L_2(1-\tilde{P}) - \delta L_2 + \boxed{bk\left((1-\mu)L_2 + (1-\mu)L_{12}/2\right)\frac{\boldsymbol{B}}{\tilde{P}}} - kL_2, \tag{1c}$$

$$\dot{\tilde{L}}_2 = \tilde{L}_2(1-\tilde{P}) - \delta\tilde{L}_2 + \boxed{bk\left(\mu L_2 + \tilde{L}_2 + \mu L_{12}/2 + \tilde{L}_{12}/2\right)\frac{\boldsymbol{B}}{\tilde{P}}} - \boxed{bk\left(L_1 + L_{12}/2 + \tilde{L}_{12}/2\right)\frac{\tilde{L}_2}{\tilde{P}}} - k\tilde{L}_2, \tag{1d}$$

$$\dot{L}_{12} = \alpha_{L_2} L_{12}(1-\tilde{P}) - \delta L_{12} + \boxed{bk\left((1-\mu)L_2 + (1-\mu)L_{12}/2\right)\frac{\boldsymbol{L_1}}{\tilde{P}}} - kL_{12}, \tag{1e}$$

$$\dot{\tilde{L}}_{12} = \tilde{L}_{12}(1-\tilde{P}) - \delta\tilde{L}_{12} + \boxed{bk\left(\mu L_2 + \tilde{L}_2 + \mu L_{12}/2 + \tilde{L}_{12}/2\right)\frac{\boldsymbol{L_1}}{\tilde{P}}} + \boxed{bk\left(L_1 + L_{12}/2 + \tilde{L}_{12}/2\right)\frac{\tilde{L}_2}{\tilde{P}}} - k\tilde{L}_{12} \tag{1f}$$

$$\dot{\tilde{P}} = \underbrace{S}_{\text{inflow}} + \underbrace{\left(B + L_1 + \tilde{L}_2 + \tilde{L}_{12} + \alpha_{L_2}(L_2 + L_{12})\right)(1-\tilde{P})}_{\text{overall growth of bacteria}} - \underbrace{\delta\tilde{P}}_{\text{dilution}} - \underbrace{k\Big(L_1 + L_2 + \tilde{L}_2 + L_{12} + \tilde{L}_{12}\Big)}_{\text{bacteria killed through lysis}} \tag{Σ1a–1f}$$

In my opinion, the red boldface multipliers $\boldsymbol{B}$, $\boldsymbol{B}$, $\boldsymbol{L_1}$, $\boldsymbol{L_1}$, in equations (1c), (1d), (1e), (1f) should appear, otherwise, the infection process is not conservative. In comparison, Equations (1)–(4) look good as the terms describing the compartmental transitions omit each other when the four equations are summed up. Namely, the compartmental transitions does not manipulate the overall number of bacteria.
