# Supplementary material for: The value of a prophage-borne defense system in phage–phage competition
Source: PLoS Comput Biol. 2026 Sep 8;22(9):e1014739. doi: 10.1371/journal.pcbi.1014739 (PMC13577574; doi:10.1371/journal.pcbi.1014739)
Supplement: Supplementary file 2 [file pcbi.1014739.s003.pdf]

Reviewer 1

REVIEW for submission PCOMPBIOL-D-26-00504 to PLOS Computational Biology

"The Value of a Prophage-Borne Defense System in Phage–Phage Competition"

by Yigal Meir and Ned S. Wingreen

The authors present a theoretical framework to study competition between temperate bacteriophages that infect the same bacterial host, with a particular focus on the evolutionary role of prophage-borne defense systems. The manuscript combines biological motivation with a relatively simple dynamical model formulated as a system of ordinary differential equations describing the interactions between susceptible bacteria and a few combinations of lysogens.

Personally, I appreciate short and concise articles, and this manuscript largely falls into that category, which made it relatively straightforward to read and review. At the same time, the presentation might benefit from a slightly more detailed discussion of the dynamical role (biological interpretation) of the individual terms in the ODE system. E.g., it would be very helpful in understanding the structure of the model if the authors were to explain the process transitions using one of the classical methods, e.g., compartmental transitions. I feel that mass action kinetics are also applicable here, as the transitions in Fig. 1B can be interpreted as chemical-like reactions.

We appreciate the reviewer's advice. The paragraph after Eqs. 1-4 has been changed accordingly.

I should also note that my own background is primarily on the mathematical side, and I am not a specialist in the biological aspects of bacteriophage systems. Nevertheless, I made an effort to follow the biological motivation and the modeling assumptions throughout the manuscript.

While going through the equations carefully, I noticed a few potential typos in Eq. (6). In Section 1, I outline this point in more detail.

Typos corrected, we thank the reviewer for pointing these out.

In addition, I did not identify previously published work by the authors that would substantially overlap with the present manuscript.

Indeed, while we have published some studies addressing competitions among phages, this is the first, and so far only work in which we consider a phage that carries its own defense system.

Minor comments and suggestions

Although I did not systematically check the manuscript from a spelling or copy-editing perspective, I have a few minor remarks. Authors may decide, at their best assessment, whether or not to accept these recommendations.

• Fig 1B. It would be convenient to emphasize that the transition in Fig 1B encode the six possible pairs of the transition from Fig 1A (an induction + an infection).

A good suggestion. We have expanded the caption to explain the schematic better, and we broke up each arrow in (B) into two arrows, to indicate that it represents the two subsequent processes in (A).

• Fig 1F. is a very fine grained vector graphics, and it requires a long time to load. Consider whether it is worth generating a high-quality rasterized image instead.

We replaced the vector graphics of Fig. 1F by an image. (We can resort back to the original if the editor thinks it is not of high enough quality.)

• It was an even longer time before I realized that ”SI Appendix” refers to ”Supporting Information.” I thought I missed to download an additional supplementary document. I advise a more straightforward reference to this section in the main text.

We have moved the equations to a Materials and Methods section.

• The lines of Eq. (6) are too long. As far as I know, the following LaTeX environment can expand the equations

along the whole width of the page:

\begin{adjustwidth}{-2.25in}{0in}column.

\begin{equation} ....

\end{equation}

\end{adjustwidth}

Done, though we prefer to leave the formatting to the journal editors.

1 Equations allowing for mutations and loss of defense systems

Figure 1: Compartmental transitions of the phage-phage competition. The infection mechanisms can be written as compartmental transitions, which preserves the overall population. here is not to challenge the overall conclusions of the manuscript, but simply to clarify a point that may help make the mathematical formulation more transparent.

We have rewritten the caption to make the model clearer.

In my opinion, the red boldface multipliers B, B, L1, L1, in equations (1c), (1d), (1e), (1f) should appear, otherwise, the infection process is not conservative. In comparison, Equations (1)–(4) look good as the terms describing the compartmental transitions omit each other when the four equations are summed up.

Corrected, thanks for pointing out the typos.

<u>Reviewer 2</u>

The authors model competition between two temperate phage variants that differ by the carriage of a costly defense system, identifying three cost-dependent regimes: dominance of the defense-carrying prophage, coexistence, and loss of the defense-carrying variant. They further extend the model by allowing mutational loss of the defense system, which eliminates the takeover regime. The manuscript also briefly considers a three-phage system, showing that non-transitive relationships can emerge.

The modeling framework is clear and, to my assessment, the results appear scientifically sound. However, I find that the main conclusions follow rather directly from general ecological and evolutionary principles governing competition between a costly trait and a trait-free variant. In particular, the three-regime, cost-dependent outcome is an expected consequence of fitness trade-offs and does not seem specific to temperate phage biology. Similarly, the additional results, including defense loss by mutation and the possibility of non-transitive interactions in a three-phage system, are largely consistent with straightforward expectations from the model structure.

While the study provides a useful formalization and adds incremental knowledge to the field, I do not

see a sufficiently strong conceptual advance or unexpected biological insight to justify publication in this journal. In my view, the manuscript would be better suited for a more specialized journal.

We appreciate the reviewer's frank assessment and are glad they find that the modeling framework to be clear and the results to be scientifically sound. Regarding the manuscript's conceptual advances/biological insights, we highlight two areas that we believe merit publication in PLoS Computational Biology (the latter including new results):

1. *Modeling advance.* While bacteria-phage competitions have been the subject of modeling studies for multiple decades, there is to our knowledge no extant modeling study of phage-phage competition involving prophage-borne defense systems. Recent experimental discoveries (e.g. current references 1-5) strongly motivate such a study. However, we found that existing modeling frameworks were not well-suited to model the long timescales characteristic of competition among slowly inducing lysogens. To this end, we introduced a novel approximation in which induction, virion burst production, and subsequent infection (or failed infection) are treated as a single instantaneous process. This approximation is well motivated by the separation of time scales between slow induction rates and these much faster processes, and allowed us to efficiently model lysogen-lysogen competitions over long times and a broad range of parameters, necessary to produce the phase diagrams in the manuscript.
2. *Biological insight.* We appreciate the reviewer's viewpoint that the qualitative results follow from the model's structure. But we highlight below several points that were not obvious from the outset (at least to us): (*i*) While it was natural to expect a transition from a regime where lysogen L1 (defenseless) is driven to extinction and one where L2 (defensive) is driven to extinction as a function of the cost of defense, it was not obvious that there would be a regime of stable coexistence of these two lysogens along with the double lysogen L12, nor that the extent of this regime would increase substantially with burst size. (*ii*) Similarly, we did not anticipate the regime of complete coexistence of all strains in the case where the defense system could be lost by mutation. (*iii*) The detailed dynamics of the non-transitive rock-paper-scissors system were also not expected by us a priori. (*iv*) Finally, in part motivated by the reviewer's desire for more unexpected insights, we extended the model to consider a lysogen carrying an abortive infection system. The required small change to the mathematical model (L2 dies upon attempted infection by an L1-type phage virion) leads to surprisingly different qualitative results. In particular, the system now displays new bistable phases in which the initial population ratios determine final steady states. These new results are reported in a new figure and corresponding subsection of Results. We thank the reviewer for their remarks, which encouraged us to push the model further to explore new territory.

Reviewer 3

Meir et al. propose a simplified mathematical framework that identifies regimes under which phage-encoded defense systems can be evolutionarily stable. The conclusions derived from the model are interesting, and the study has the potential to make a meaningful contribution to the field. I have some minor comments that should be addressed to improve the manuscript's readability, organization, and thoroughness.

L20f: The Introduction section would benefit from a more thorough literature review of mathematical models developed to address similar questions. It can help position this study against prior modeling efforts.

We appreciate the reviewer's suggestion, and have added the following text and citations to the Introduction: "Bacteria–phage population dynamics have long been modeled using resource-explicit

predator–prey or chemostat frameworks, including the classic work of Levin, Stewart, and Chao on lytic phages [10] and a later extension to study the advantages of lysogeny [11].”

However, to our knowledge, ours is the first modeling study that directly addresses phage-borne defense systems against unrelated phages.

L38f: There is no dedicated “Methods and Materials” section, and key descriptions of the models (e.g., the two-phage system, system with mutation, and the governing equations) currently appear within the Results section. I suggest moving model formulation and equation descriptions into a Methods and Materials section, which would help separate model setup from interpretation of results.

The suggestions is appreciated. Except for the first set of dimensionless equations, where we present the model, we have moved all other equations to a Materials and Methods section.

The Results section would also benefit from clearer subsections organized around the main questions being addressed or the main takeaways of each figure.

We appreciate the suggestion and have added subsection headings within the Results section.

L44f: Assumptions (1) and (2): Do other mathematical models developed in a similar context also adopt these assumptions?

Assumption (1) is a standard chemostat formulation. Assumption (2), on the other hand, is a unique ingredient in this simulation, as to model the time scales of induction it is both reasonable and advantageous to assume infection is instantaneous. We now explicitly point out this original assumption above Eq. 1-4: “We do not track free phage particles, instead assuming that these rapidly find bacterial targets. To our knowledge, this useful approximation for modeling lysogen population dynamics is new to the field.“

Figure 1A-B: It took me some time to understand what diagrams mean and how they connect to the equations. In the current design, blue and red circles inside the orange blobs represent lysogens’ identity while blue and red circles outside the orange blobs represent released phage virions with and without a defense system. The labels next to blue and red circles at the bottom of Panel(A) are somewhat confusing. It would also help to label the reaction/process rates directly on the schematic.

We appreciate the suggestion and have added a more detailed explanation in the caption, and changed the labels to “Phage/prophage” .

L50f: In Eq. (3), the absence of an invasion loss term analogous to that in Eq. (2) (the third term in Eq.(2)) appears to encode the protection conferred by the defense system. It may help to state this explicitly when introducing the equations. On a similar note, the authors should explain the biological meaning of each term in the equations, rather than only introducing the reaction-rate parameters.

Done – see our response Reviewer 1.

L55: It’s assumed that non-defense prophage carriage has no cost. This assumption is important for interpreting the competition between L1 and L2, but it is not fully justified. The authors should

explain why this assumption is reasonable, or discuss whether the results are sensitive to allowing a small fitness cost for L1.

There are limited studies of the cost of carrying a prophage that, like L1, has a mechanism for superinfection exclusion. One notable exception is the work of Bondy-Denomy et al. (ISME J 2016) in which it was reported that a *P. aeruginosa* prophage-mediated surface-modification mechanism was able to provide strong resistance to superinfection, yet, within measurement accuracy, was cost-free in both soil and *C. elegans* infection models. So, while we believe our assumption that the cost to the host of such a prophage can be small, we appreciate the reviewer's concern regarding this assumption. To that end, we repeated our simulations with a modest cost to the L1 phage (growth rate reduced to 90% of that of the susceptible bacteria) and found no qualitative changes to our results. We now report this below Eqs. 1-4: "(we also tested an intermediate lower growth rate for the defenseless lysogen, which did not lead to any qualitative changes in the results)"

L63-65: what is the rationale for selecting the three alpha_L2 levels? How do you determine that alpha_L2 = 0.8 is relatively high for the growth rate?

The three alpha_L2 levels are chosen to be in the 3 different regimes, and the terminology refers to the order of these values.

L70-71: I understand authors are trying to say a successful defense may remove the very threat that made the defense beneficial. But it sounds more like evolutionary intuition than a direct result emerging from the model. Because it immediately follows "Fig. 1F suggests…," readers may interpret this as a continuation of the interpretation of Fig. 1, rather than a conceptual motivation for introducing defense loss caused by mutation in Fig. 2.

To avoid possible confusion, we have softened the wording near the beginning of this paragraph to make it clearer that we are considering a possibility, not a direct result of the model.

Figures 2 C and D: Lines with colors other than green and the mixture of green and red are not labeled.

We now state in the caption that the line colors in the legend are in addition to the line colors in the legend of Fig. 1D.

L80-83: kind of circling back to my previous comment, how were the parameter values associated with "small" versus "high" cost determined? For example, αlpha_L2 =0.90 and 0.95 are only marginally different, and without a clearer baseline it is difficult to interpret what constitutes low or high cost.

As noted above, the terms "smallest", "largest" simply refer to the numerical order of these parameter values.

In addition, what criteria were used to determine "coexistence" and "symmetrical coexistence," other than visual inspection of the trajectories? Was there a quantitative definition or threshold used to classify these outcomes in the simulations? Relatedly, what is meant precisely by "symmetrical coexistence"?

Since the simulations are deterministic, without noise, it is mathematically unambiguous when an outcome is coexistence or extinction. We use the term "symmetrical coexistence" only in the case where the two competing lysogens have identical characteristics – in this case there is nothing in the equations that separate the two lysogens, so the outcome is exactly symmetric, as seen in Fig. 1D.

We have modified a sentence to clarify that point: "For a higher cost, the two lysogens without defense systems, L1 and ˜L2, coexist symmetrically (Fig. 3D), as they have identical characteristics."

L86: The conclusion that L2 outcompetes L1, and later that the two coexist as effectively identical competitors, appears to rely on the assumption that carrying a prophage without a defense system imposes negligible fitness cost. Would these conclusions still hold if L1 incurred a non-zero cost?

There are two points here. First, as discussed above, a non-zero cost to L1 of carrying a prophage shifts the phase boundaries in Fig. 1F, 2A, and 3E, but does not qualitatively change the results. Second, the mutant version of L2 that loses its defense system still carries a prophage so it should have the same growth rate as L1, and so the two should co-exist symmetrically even if there is a non-zero cost to carrying a prophage.

L90: The authors could consider placing the model formulation of the three-phage system in the Supplementary Information.

We have added the relevant equation to the new "Materials and Methods" section.
