# Supplementary material for: The value of a prophage-borne defense system in phage–phage competition
Source: PLoS Comput Biol. 2026 Sep 8;22(9):e1014739. doi: 10.1371/journal.pcbi.1014739 (PMC13577574; doi:10.1371/journal.pcbi.1014739)
Supplement: Supplementary file 3 [file pcbi.1014739.s004.pdf]

Dear Editors,

Thank you for obtaining the second reports from the three reviewers, and we are glad that you consider the manuscript to have merit. We are gratified that Reviewers #1 and #3 were satisfied with the revisions, and that Reviewer #2 found that “the inclusion of abortive infection leads to qualitatively different behavior, which goes beyond the standard expectations derived from general ecological trade-off theory”. That reviewer encouraged us “to more explicitly analyze and articulate the underlying mechanism that gives rise to bistability”. This is an excellent suggestion, and we have done exactly this in the revised manuscript. We have also taken to heart the editorial request to discuss the limitations of the current study by extending that portion of the manuscript.

Our specific revisions to address these points are described in detail below, with our responses in blue and revised text in red.

We hope that with these improvements, the manuscript may now be deemed suitable for publication in PLOS Computational Biology.

Sincerely,
Yigal Meir & Ned Wingreen

Reviewer #2: I appreciate the authors’ thoughtful response and their effort to extend the manuscript by incorporating the case of abortive infection. This is a welcome addition. In particular, I agree that the inclusion of abortive infection leads to qualitatively different behavior, which goes beyond the standard expectations derived from general ecological trade-off theory. However, the manuscript still falls short in explaining why such differences arise. The presentation remains largely descriptive. Bistability is typically associated with the presence of positive feedback, yet the authors do not identify where such feedback emerges/being strengthened in their model when abortive infection is introduced. Relatedly, it remains unclear why this feature is absent in the baseline model.

Without a clear mechanistic explanation, the new results remain insufficiently interpreted. As a result, this extension, while intriguing, does not yet provide a fully developed biological insight. I encourage the authors to more explicitly analyze and articulate the underlying mechanism that gives rise to bistability and to contrast it directly with structure of the original model.

This is an excellent suggestion which motivated us to carefully consider the origin of bistability in the case of abortive infection. We found an insightful mathematical approach by considering the dynamics of the difference between the populations of L_1 and L_2 lysogens in the limit of equal growth rates (see Eq. 5). Specifically, we found that in the bistable regime there is instability about the condition $L_1 = L_2$. Along with a careful consideration of the ability of each strain to invade the other in this regime, we believe we have provided some helpful mechanistic insight into the origin of bistability in this case. This material is contained in the new paragraph surrounding Eq. 5.

Regarding data and code availability, Reviewer #2 also wrote: There is a link to a website, but the content seems to be limited to the model and data for Fig. 1 only. The readme is not properly documented either.

In fact, the annotated Mathematica code for all four figures was present on the website. To clarify that the code is fully annotated, we have revised the readme file to indicate this.

Finally, regarding the editorial request to discuss limitations of the current study, we have added text to the corresponding paragraph in the Discussion and Conclusion section which now reads:

Some natural extensions of our model would include competition between defensive temperate phages and lytic phages or phages carrying counter-defense systems. We have also assumed that the defense system is absolute, whereas defenses may only be partial or may be regulated, e.g. by quorum sensing [19,20]. In natural settings, the competition among lysogens may also be spatially structured (our lysogen-only representation may still be useful for spatiotemporal modeling under the assumption that free virions quickly find targets). These scenarios and settings will quantitatively change competitive outcomes, but are not expected to change the overall advantage of phage-borne defenses. Indeed, a recent theoretical study of competition between superinfection-exclusion-positive or -negative lytic phages came to a similar conclusion regarding the substantial value of such a phage-borne defense [21].
