# Supplementary material for: The value of a prophage-borne defense system in phage–phage competition
Source: PLoS Comput Biol. 2026 Sep 8;22(9):e1014739. doi: 10.1371/journal.pcbi.1014739 (PMC13577574; doi:10.1371/journal.pcbi.1014739)
Supplement: Peer review history

# Peer review: Decision Letter 0

**DOI:** 10.1371/journal.pcbi.1014739.r001

**Authors:** Amber Smith, Boyang Ji

28 Apr 2026

The Value of a Prophage-Borne Defense System in Phage–Phage Competition

PLOS Computational Biology

Dear Dr. Wingreen,

Thank you for submitting your manuscript to PLOS Computational Biology. After careful consideration, we feel that it has merit but does not fully meet PLOS Computational Biology's publication criteria as it currently stands. Therefore, we invite you to submit a revised version of the manuscript that addresses the points raised during the review process.

Please submit your revised manuscript by Jun 28 2026 11:59PM. If you will need more time than this to complete your revisions, please reply to this message or contact the journal office at ploscompbiol@plos.org. When you're ready to submit your revision, log on to https://www.editorialmanager.com/pcompbiol/ and select the 'Submissions Needing Revision' folder to locate your manuscript file.

Please include the following items when submitting your revised manuscript:

* A letter that responds to each point raised by the editor and reviewer(s). You should upload this letter as a separate file labeled 'Response to Reviewers'. This file does not need to include responses to formatting updates and technical items listed in the 'Journal Requirements' section below.

* A marked-up copy of your manuscript that highlights changes made to the original version. You should upload this as a separate file labeled 'Revised Manuscript with Track Changes'.

* An unmarked version of your revised paper without tracked changes. You should upload this as a separate file labeled 'Manuscript'.

If you would like to make changes to your financial disclosure, competing interests statement, or data availability statement, please make these updates within the submission form at the time of resubmission. Guidelines for resubmitting your figure files are available below the reviewer comments at the end of this letter

We look forward to receiving your revised manuscript.

Kind regards,

Boyang Ji, Ph.D.

Academic Editor

PLOS Computational Biology

Amber Smith

Section Editor

PLOS Computational Biology

**Journal Requirements:**

1) Please ensure that the CRediT author contributions listed for every co-author are completed accurately and in full.

At this stage, the following Authors/Authors require contributions: Yigal Meir, and Ned S. Wingreen. Please ensure that the full contributions of each author are acknowledged in the "Add/Edit/Remove Authors" section of our submission form.

The list of CRediT author contributions may be found here: https://journals.plos.org/ploscompbiol/s/authorship#loc-author-contributions

2) We ask that a manuscript source file is provided at Revision. Please upload your manuscript file as a .doc, .docx, .rtf or .tex. If you are providing a .tex file, please upload it under the item type u2018LaTeX Source Fileu2019 and leave your .pdf version as the item type u2018Manuscriptu2019.

3) Please upload all main figures as separate Figure files in .tif or .eps format. For more information about how to convert and format your figure files please see our guidelines:

https://journals.plos.org/ploscompbiol/s/figures

4) In the online submission form, you indicated that "Code can be made available by request". All PLOS journals now require all data underlying the findings described in their manuscript to be freely available to other researchers, either

1. In a public repository

2. Within the manuscript itself

3. Uploaded as supplementary information.

This policy applies to all data except where public deposition would breach compliance with the protocol approved by your research ethics board. If your data cannot be made publicly available for ethical or legal reasons (e.g., public availability would compromise patient privacy), please explain your reasons by return email and your exemption request will be escalated to the editor for approval. Your exemption request will be handled independently and will not hold up the peer review process, but will need to be resolved should your manuscript be accepted for publication. One of the Editorial team will then be in touch if there are any issues.

**Reviewers' comments:**

Reviewer's Responses to Questions

**Comments to the Authors:**

**Please note here if the review is uploaded as an attachment.**

Reviewer #1: See, the attached PDF.

Reviewer #2: The authors model competition between two temperate phage variants that differ by the carriage of a costly defense system, identifying three cost-dependent regimes: dominance of the defense-carrying prophage, coexistence, and loss of the defense-carrying variant. They further extend the model by allowing mutational loss of the defense system, which eliminates the takeover regime. The manuscript also briefly considers a three-phage system, showing that non-transitive relationships can emerge.

The modeling framework is clear and, to my assessment, the results appear scientifically sound. However, I find that the main conclusions follow rather directly from general ecological and evolutionary principles governing competition between a costly trait and a trait-free variant. In particular, the three-regime, cost-dependent outcome is an expected consequence of fitness trade-offs and does not seem specific to temperate phage biology. Similarly, the additional results, including defense loss by mutation and the possibility of non-transitive interactions in a three-phage system, are largely consistent with straightforward expectations from the model structure.

While the study provides a useful formalization and adds incremental knowledge to the field, I do not see a sufficiently strong conceptual advance or unexpected biological insight to justify publication in this journal. In my view, the manuscript would be better suited for a more specialized journal.

Reviewer #3: Meir et al. propose a simplified mathematical framework that identifies regimes under which phage-encoded defense systems can be evolutionarily stable. The conclusions derived from the model are interesting, and the study has the potential to make a meaningful contribution to the field. I have some minor comments that should be addressed to improve the manuscript’s readability, organization, and thoroughness.

L20f: The Introduction section would benefit from a more thorough literature review of mathematical models developed to address similar questions. It can help position this study against prior modeling efforts.

L38f: There is no dedicated “Methods and Materials” section, and key descriptions of the models (e.g., the two-phage system, system with mutation, and the governing equations) currently appear within the Results section. I suggest moving model formulation and equation descriptions into a Methods and Materials section, which would help separate model setup from interpretation of results.

The Results section would also benefit from clearer subsections organized around the main questions being addressed or the main takeaways of each figure.

L44f: Assumptions (1) and (2): Do other mathematical models developed in a similar context also adopt these assumptions?

Figure 1A-B: It took me some time to understand what diagrams mean and how they connect to the equations. In the current design, blue and red circles inside the orange blobs represent lysogens’ identity while blue and red circles outside the orange blobs represent released phage virions with and without a defense system. The labels next to blue and red circles at the bottom of Panel(A) are somewhat confusing. It would also help to label the reaction/process rates directly on the schematic.

L50f: In Eq. (3), the absence of an invasion loss term analogous to that in Eq. (2) (the third term in Eq.(2)) appears to encode the protection conferred by the defense system. It may help to state this explicitly when introducing the equations. On a similar note, the authors should explain the biological meaning of each term in the equations, rather than only introducing the reaction-rate parameters.

L55: It’s assumed that non-defense prophage carriage has no cost. This assumption is important for interpreting the competition between L1 and L2, but it is not fully justified. The authors should explain why this assumption is reasonable, or discuss whether the results are sensitive to allowing a small fitness cost for L1.

L63-65: what is the rationale for selecting the three alpha_L2 levels? How do you determine that alpha_L2 = 0.8 is relatively high for the growth rate?

L70-71: I understand authors are trying to say a successful defense may remove the very threat that made the defense beneficial. But it sounds more like evolutionary intuition than a direct result emerging from the model. Because it immediately follows “Fig. 1F suggests…,” readers may interpret this as a continuation of the interpretation of Fig. 1, rather than a conceptual motivation for introducing defense loss caused by mutation in Fig. 2.

Figures 2 C and D: Lines with colors other than green and the mixture of green and red are not labeled.

L80-83: kind of circling back to my previous comment, how were the parameter values associated with “small” versus “high” cost determined? For example, αlpha_L2 =0.90 and 0.95 are only marginally different, and without a clearer baseline it is difficult to interpret what constitutes low or high cost. In addition, what criteria were used to determine “coexistence” and “symmetrical coexistence,” other than visual inspection of the trajectories? Was there a quantitative definition or threshold used to classify these outcomes in the simulations? Relatedly, what is meant precisely by “symmetrical coexistence”?

L86: The conclusion that L2 outcompetes L1, and later that the two coexist as effectively identical competitors, appears to rely on the assumption that carrying a prophage without a defense system imposes negligible fitness cost. Would these conclusions still hold if L1 incurred a non-zero cost?

L90: The authors could consider placing the model formulation of the three-phage system in the Supplementary Information.

**********

**Have the authors made all data and (if applicable) computational code underlying the findings in their manuscript fully available?**

The PLOS Data policy requires authors to make all data and code underlying the findings described in their manuscript fully available without restriction, with rare exception (please refer to the Data Availability Statement in the manuscript PDF file). The data and code should be provided as part of the manuscript or its supporting information, or deposited to a public repository. For example, in addition to summary statistics, the data points behind means, medians and variance measures should be available. If there are restrictions on publicly sharing data or code —e.g. participant privacy or use of data from a third party—those must be specified.

Reviewer #1: None

Reviewer #2: **No:** It says "code can be made available by request", but it is not publicly available.

Reviewer #3: **No:**

**********

PLOS authors have the option to publish the peer review history of their article (what does this mean?). If published, this will include your full peer review and any attached files.

If you choose “no”, your identity will remain anonymous but your review may still be made public.

**Do you want your identity to be public for this peer review?**  For information about this choice, including consent withdrawal, please see our Privacy Policy.

Reviewer #1: No

Reviewer #2: No

Reviewer #3: No

[NOTE: If reviewer comments were submitted as an attachment file, they will be attached to this email and accessible via the submission site. Please log into your account, locate the manuscript record, and check for the action link "View Attachments". If this link does not appear, there are no attachment files.]

**Figure resubmission:**

After uploading your figures to PLOS’s NAAS tool - https://ngplosjournals.pagemajik.ai/artanalysis, NAAS will process the files provided and display the results in the "Uploaded Files" section of the page as the processing is complete. If the uploaded figures meet our requirements (or NAAS is able to fix the files to meet our requirements), the figure will be marked as "fixed" above. If NAAS is unable to fix the files, a red "failed" label will appear above. When NAAS has confirmed that the figure files meet our requirements, please download the file via the download option, and include these NAAS processed figure files when submitting your revised manuscript.

**Reproducibility:**

?>

Attachment Submitted filename: Review.pdf

# Author comment: Author response to Decision Letter 1

**DOI:** 10.1371/journal.pcbi.1014739.r002

9 Jun 2026

Attachment Submitted filename: Response to reviews.pdf

# Peer review: Decision Letter 1

**DOI:** 10.1371/journal.pcbi.1014739.r003

**Authors:** Amber Smith, Boyang Ji

21 Jul 2026

PCOMPBIOL-D-26-00504R1

The Value of a Prophage-Borne Defense System in Phage–Phage Competition

PLOS Computational Biology

Dear Dr. Wingreen,

Thank you for submitting your manuscript to PLOS Computational Biology. After careful consideration, we feel that it has merit but does not fully meet PLOS Computational Biology's publication criteria as it currently stands. Therefore, we invite you to submit a revised version of the manuscript that addresses the points raised during the review process.

Please submit your revised manuscript by Sep 20 2026 11:59PM. If you will need more time than this to complete your revisions, please reply to this message or contact the journal office at ploscompbiol@plos.org. When you're ready to submit your revision, log on to https://www.editorialmanager.com/pcompbiol/ and select the 'Submissions Needing Revision' folder to locate your manuscript file.

Please include the following items when submitting your revised manuscript:

* A letter that responds to each point raised by the editor and reviewer(s). You should upload this letter as a separate file labeled 'Response to Reviewers'. This file does not need to include responses to formatting updates and technical items listed in the 'Journal Requirements' section below.

* A marked-up copy of your manuscript that highlights changes made to the original version. You should upload this as a separate file labeled 'Revised Manuscript with Track Changes'.

* An unmarked version of your revised paper without tracked changes. You should upload this as a separate file labeled 'Manuscript'.

If you would like to make changes to your financial disclosure, competing interests statement, or data availability statement, please make these updates within the submission form at the time of resubmission. Guidelines for resubmitting your figure files are available below the reviewer comments at the end of this letter.

As the corresponding author, your ORCID iD is verified in the submission system and will appear in the published article. PLOS supports the use of ORCID, and we encourage all coauthors to register for an ORCID iD and use it as well. Please encourage your coauthors to verify their ORCID iD within the submission system before final acceptance, as unverified ORCID iDs will not appear in the published article. Only the individual author can complete the verification step; PLOS staff cannot verify ORCID iDs on behalf of authors.

We look forward to receiving your revised manuscript.

Kind regards,

Boyang Ji, Ph.D.

Academic Editor

PLOS Computational Biology

Amber Smith

Section Editor

PLOS Computational Biology

**Additional Editor Comments (if provided):**

Most of the comments from the previous round of review have been adequately addressed. Reviewer 2 raised an interesting question regarding the underlying reasons for the observed bistability in this system. It would be valuable for the authors to discuss this issue further, along with the limitations of the current study. In addition, the associated website could be further improved for more details.

**Journal Requirements:**

If the reviewer comments include a recommendation to cite specific previously published works, please review and evaluate these publications to determine whether they are relevant and should be cited. There is no requirement to cite these works unless the editor has indicated otherwise.

1) We noticed that you used the phrase 'not shown' in the manuscript. We do not allow these references, as the PLOS data access policy requires that all data be either published with the manuscript or made available in a publicly accessible database. Please amend the supplementary material to include the referenced data or remove the references.

**Reviewers' comments:**

Reviewer's Responses to Questions

**Comments to the Authors:**

**Please note here if the review is uploaded as an attachment.**

Reviewer #1: The Authors addressed all my comments and concerns. A do not have any further comments.

Reviewer #2: I appreciate the authors’ thoughtful response and their effort to extend the manuscript by incorporating the case of abortive infection. This is a welcome addition. In particular, I agree that the inclusion of abortive infection leads to qualitatively different behavior, which goes beyond the standard expectations derived from general ecological trade-off theory.

However, the manuscript still falls short in explaining why such differences arise. The presentation remains largely descriptive. Bistability is typically associated with the presence of positive feedback, yet the authors do not identify where such feedback emerges/being strengthened in their model when abortive infection is introduced. Relatedly, it remains unclear why this feature is absent in the baseline model.

Without a clear mechanistic explanation, the new results remain insufficiently interpreted. As a result, this extension, while intriguing, does not yet provide a fully developed biological insight. I encourage the authors to more explicitly analyze and articulate the underlying mechanism that gives rise to bistability and to contrast it directly with structure of the original model.

Reviewer #3: I thank the authors for their revisions. They have addressed all of my previous comments.

**********

**Have the authors made all data and (if applicable) computational code underlying the findings in their manuscript fully available?**

The PLOS Data policy requires authors to make all data and code underlying the findings described in their manuscript fully available without restriction, with rare exception (please refer to the Data Availability Statement in the manuscript PDF file). The data and code should be provided as part of the manuscript or its supporting information, or deposited to a public repository. For example, in addition to summary statistics, the data points behind means, medians and variance measures should be available. If there are restrictions on publicly sharing data or code —e.g. participant privacy or use of data from a third party—those must be specified.

Reviewer #1: None

Reviewer #2: **No:** There is a link to a website, but the content seems to be limited to the model and data for Fig. 1 only. The readme is not properly documented either.

Reviewer #3: Yes

**********

PLOS authors have the option to publish the peer review history of their article (what does this mean?). If published, this will include your full peer review and any attached files.

If you choose “no”, your identity will remain anonymous but your review may still be made public.

**Do you want your identity to be public for this peer review?**  For information about this choice, including consent withdrawal, please see our Privacy Policy.

Reviewer #1: No

Reviewer #2: No

Reviewer #3: No

[NOTE: If reviewer comments were submitted as an attachment file, they will be attached to this email and accessible via the submission site. Please log into your account, locate the manuscript record, and check for the action link "View Attachments". If this link does not appear, there are no attachment files.]

**Figure resubmission:**

**Reproducibility:**

To enhance the reproducibility of your results, we recommend that authors of applicable studies deposit laboratory protocols in protocols.io, where a protocol can be assigned its own identifier (DOI) such that it can be cited independently in the future. Additionally, PLOS ONE offers an option to publish peer-reviewed clinical study protocols. Read more information on sharing protocols at https://plos.org/protocols?utm_medium=editorial-email&utm_source=authorletters&utm_campaign=protocols

# Author comment: Author response to Decision Letter 2

**DOI:** 10.1371/journal.pcbi.1014739.r004

18 Aug 2026

Attachment Submitted filename: Response to reviewers.pdf

# Editor report: Decision Letter 2

**DOI:** 10.1371/journal.pcbi.1014739.r005

**Authors:** Amber Smith, Boyang Ji

19 Aug 2026

Dear Prof. Wingreen,

We are pleased to inform you that your manuscript 'The Value of a Prophage-Borne Defense System in Phage–Phage Competition' has been provisionally accepted for publication in PLOS Computational Biology.

Before your manuscript can be formally accepted you will need to complete some formatting changes, which you will receive in a follow up email. A member of our team will be in touch with a set of requests.

Please note that your manuscript will not be scheduled for publication until you have made the required changes, so a swift response is appreciated.

IMPORTANT: The editorial review process is now complete. PLOS will only permit corrections to spelling, formatting or significant scientific errors from this point onwards. Requests for major changes, or any which affect the scientific understanding of your work, will cause delays to the publication date of your manuscript.

Should you, your institution's press office or the journal office choose to press release your paper, you will automatically be opted out of early publication. We ask that you notify us now if you or your institution is planning to press release the article. All press must be co-ordinated with PLOS.

Thank you again for supporting Open Access publishing; we are looking forward to publishing your work in PLOS Computational Biology.

Best regards,

Boyang Ji, Ph.D.

Academic Editor

PLOS Computational Biology

Amber Smith

Section Editor

PLOS Computational Biology

***********************************************************

# Editor report: Acceptance letter

**DOI:** 10.1371/journal.pcbi.1014739.r006

**Authors:** Amber Smith, Boyang Ji

PCOMPBIOL-D-26-00504R2

The Value of a Prophage-Borne Defense System in Phage–Phage Competition

Dear Dr Wingreen,

I am pleased to inform you that your manuscript has been formally accepted for publication in PLOS Computational Biology. Your manuscript is now with our production department and you will be notified of the publication date in due course.

The corresponding author will soon be receiving a typeset proof for review, to ensure errors have not been introduced during production. Please review the PDF proof of your manuscript carefully, as this is the last chance to correct any errors. Please note that major changes, or those which affect the scientific understanding of the work, will likely cause delays to the publication date of your manuscript.

Soon after your final files are uploaded, unless you have opted out, the early version of your manuscript will be published online. The date of the early version will be your article's publication date. The final article will be published to the same URL, and all versions of the paper will be accessible to readers.

For Research, Software, and Methods articles, you will receive an invoice from PLOS for your publication fee after your manuscript has reached the completed accept phase. If you receive an email requesting payment before acceptance or for any other service, this may be a phishing scheme. Learn how to identify phishing emails and protect your accounts at https://explore.plos.org/phishing.

Thank you again for supporting PLOS Computational Biology and open-access publishing. We are looking forward to publishing your work!

With kind regards,

Anitha Samidurai

PLOS Computational Biology | Carlyle House, Carlyle Road, Cambridge CB4 3DN | United Kingdom ploscompbiol@plos.org | Phone +44 (0) 1223-442824 | ploscompbiol.org | @PLOSCompBiol
